# Pathogen surveillance in the informal settlement, Kibera, Kenya, using a metagenomics approach

**DOI:** 10.1371/journal.pone.0222531

**Published:** 2019-10-10

**Authors:** Rene S. Hendriksen, Oksana Lukjancenko, Patrick Munk, Mathis H. Hjelmsø, Jennifer R. Verani, Eric Ng’eno, Godfrey Bigogo, Samuel Kiplangat, Traoré Oumar, Lasse Bergmark, Timo Röder, John C. Neatherlin, Onyango Clayton, Tine Hald, Susanne Karlsmose, Sünje J. Pamp, Barry Fields, Joel M. Montgomery, Frank M. Aarestrup

**Affiliations:** 1 National Food Institute, WHO Collaborating Center for Antimicrobial Resistance in Foodborne Pathogens and Genomics and European Union Reference Laboratory for Antimicrobial Resistance, Technical University of Denmark, Kgs. Lyngby, Denmark; 2 Division of Global Health Protection, Center for Global Health, Centers for Disease Control and Prevention, Nairobi, Kenya; 3 Center for Global Health, Centers for Disease Control and Prevention, Atlanta, GA, United States of America; 4 Kenya Medical Research Institute, Center for Global Health Research (KEMRI-CGHR), Nairobi, Kenya; Nanjing University, CHINA

## Abstract

**Background:**

Worldwide, the number of emerging and re-emerging infectious diseases is increasing, highlighting the importance of global disease pathogen surveillance. Traditional population-based methods may fail to capture important events, particularly in settings with limited access to health care, such as urban informal settlements. In such environments, a mixture of surface water runoff and human feces containing pathogenic microorganisms could be used as a surveillance surrogate.

**Method:**

We conducted a temporal metagenomic analysis of urban sewage from Kibera, an urban informal settlement in Nairobi, Kenya, to detect and quantify bacterial and associated antimicrobial resistance (AMR) determinants, viral and parasitic pathogens. Data were examined in conjunction with data from ongoing clinical infectious disease surveillance.

**Results:**

A large variation of read abundances related to bacteria, viruses, and parasites of medical importance, as well as bacterial associated antimicrobial resistance genes over time were detected. Significant increased abundances were observed for a number of bacterial pathogens coinciding with higher abundances of AMR genes. *Vibrio cholerae* as well as rotavirus A, among other virus peaked in several weeks during the study period whereas *Cryptosporidium* spp. and *Giardia* spp, varied more over time.

**Conclusion:**

The metagenomic surveillance approach for monitoring circulating pathogens in sewage was able to detect putative pathogen and resistance loads in an urban informal settlement. Thus, valuable if generated in real time to serve as a comprehensive infectious disease agent surveillance system with the potential to guide disease prevention and treatment. The approach may lead to a paradigm shift in conducting real-time global genomics-based surveillance in settings with limited access to health care.

## Introduction

Globally, infectious diseases are responsible for approximately 22% of all human deaths [1] and cause a substantial burden on health systems. Rapid detection of outbreaks, known pathogens or emerging novel pathogens is critical for the prevention and control of infectious diseases. The proportion of deaths in urban settlements due to infectious diseases, inadequate health care access, lower socioeconomic status and malnutrition is generally higher in low and middle income settings, where, among many other factors, an increasing proportion of the world’s population resides [1,2]. However, conducting infectious disease surveillance among urban settlement dwellers using conventional disease surveillance methods can be challenging and costly due to the dense settlements, poor sanitation, and inadequate health care access. Thus, novel local and global surveillance systems for detection and response in such settings are needed to improve containment and control of infectious diseases [3].

Sewage has been used previously for the surveillance of selected infectious disease agents, including poliovirus, hepatitis viruses A and E, non-polio enteroviruses, norovirus, parechovirus, and astrovirus [4–6]. However, such studies have only focused on a single or a limited number of the possible pathogens found in sewage. Despite metagenomics being in its infancy, several recent studies have shown the feasibility of this methodology to identify and quantify a wide range of bacteria, viruses, and antimicrobial resistance (AMR) genes from complex samples such as sewage and wastewater [5,7–12].

In this study, we used metagenomic sequencing of urban sewage to monitor the presence of pathogens and bacterial AMR genes in Kibera, an urban informal settlement in Nairobi, Kenya, and examined the findings in conjunction with other ongoing disease surveillance work in the same area.

## Materials and methods

### Ethics

This study was conducted in accordance with the Danish Act on scientific ethical treatment of health research (Journal no.: H-14013582) and fulfills the requirements of the Nagoya Protocol. Data collection in the Population-Based Infectious Disease Surveillance (PBIDS) system is approved by institutional review bodies of Kenya Medical Research Institute (KEMRI) and US Centers for Disease Control and Prevention (CDC).

Written informed consent is obtained from heads of households for their household members to participate in PBIDS. Household member’s ≥18 years are free decline participation in the surveillance. Additional written informed consent is obtained from participant (or parent/guardian) before blood and/or stool sample collection at the clinic. No additional approval was required for collection of the sewage samples. The samples were collected from open sewage runoffs draining the study area, with verbal permission of household heads who were living near the collection points.

### Study setting and collection of surveillance data

**Fig 1:**
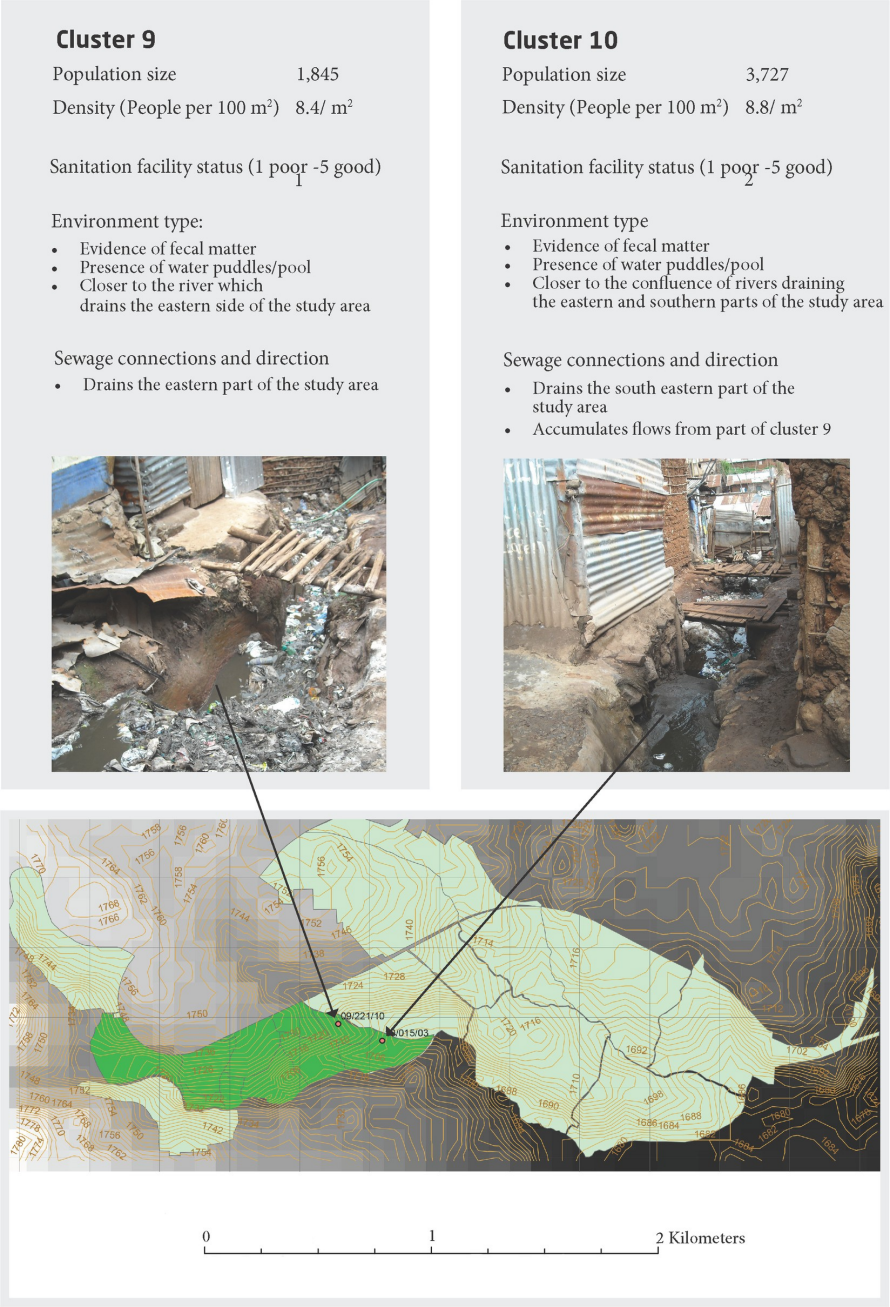
Geographical overview and location and description of the residence clusters 9 and 10 of the urban slum city of Kibera, Nairobi, Kenya. Sampling points marked with a red circle, brown lines indicate hill contours, dark green mark the PBIDS, black lines separate the residence clusters, light green mark the urban slum city. Photograph provided by the author Eric Ngeno.

Kibera is one of the largest informal settlements in East Africa with a population size ranging between 250,000–500,000 individuals (Kenya National Bureau of Statistics, The 2009 Kenya Population and Housing Census Results. 2010: Nairobi, Kenya. p. 34). The surveillance is conducted in two (Gatwekera and Soweto) out of twelve villages in Kibera. The area covers approximately 0.4 km2 and is densely populated (~70,000 persons/ km2) with high burden of infectious diseases [2,13,14]. Human fecal waste from households' latrines in the study area, flow into a network of ditches draining the area. Samples were collected at points of confluence of the drainage ditches, in two areas with the highest surface flow accumulation; cluster 9 (latitude/ longitude: -1.314199/ 36.78492, altitude 1722.55) and cluster 10 (latitude/ longitude: -1.314704/ 36.78666, altitude 1722.55). There are ten geographic units referred to as clusters in the study area (Fig 1) [15].

Since 2005, CDC and KEMRI have jointly operated the PBIDS system [2]. Household morbidity and health care usage data were collected every two weeks through home visits. Members with fever, respiratory illness or diarrhea during the home visits were advised to seek care at a centrally located Tabitha clinic (located within a ~1Km radius of all PBIDs households.) which offered free medical care for acute illnesses. Still, the’ participants can freely seek care at other private and public health care facilities, chemists/pharmacies, drug shops as well as traditional healers. At the clinic patients with acute febrile illness (AFI) defined as measured axillary temperature ≥38.0°C, respiratory syndrome defined as cough or difficult breathing plus one of IMCI danger signs or diarrhea defined as reported ≥3 loose stools in 24 hours [2], had their blood and/or stool samples collected for testing by culture methods [16][13].

For more details, see S1 File.

### Sample collections, storage and shipment

Sewage samples were collected each Monday and Wednesday. 500 mL of sewage was collected from each site during the study period (June 16 to August 26, 2014), typically a ‘dry season’ in Nairobi, resulting in a total of 42 samples [15] (Fig 1).

(S1 File, Fig 1). Collected samples were kept in cooler boxes and transported to a KEMRI laboratory in the study area within 2 hours of collection. At KEMRI laboratory the samples were stored with no presentation at -80 C° and further shipped frozen without coolers in batches to the Technical University in Denmark for DNA extraction and downstream metagenomics analysis. All samples arrived still frozen to Technical University in Denmark.

Without the knowledge of the authors responsible for the analysis, samples taken at both clusters 9 and 10 in week 28 were spiked with a 1-μl culture of *Salmonella enterica* serovar Typhi (*S*. Typhi) to test the sensitivity of the sewage metagenomics approach.

### Genomics

#### DNA and RNA extraction and whole community sequencing

Sewage samples were centrifuged at 8,000g for 30 min. The pellet was tested for bacterial and parasitic DNA, and the supernatant was extracted for DNA and RNA viruses. Genomic DNA (from bacteria, parasites and DNA viruses) was extracted from the samples using the QIAamp Fast DNA Stool mini kit as previously described [17]. Viral RNA and DNA were co-extracted using the Nucleospin RNA XS kit. The DNA- and RNA-based samples were sequenced using Illumina HiSeq (bacterial and parasitic DNA) and MiSeq (DNA and RNA viruses). Initially, trimming and removal of adaptor sequences was done using cutadapt [18]with settings for minimum read length being 30 bp and a minimum Phred quality score of 30, to trim low-quality reads before adaptor removal (cutadapt parameter—quality-cutoff). Raw sequence data have been submitted to the European Nucleotide Archive under study accession no.: PRJEB13833.

#### Metagenomics analysis

Bacteria, viruses, parasites, and AMR genes within the samples were identified and quantified using MGmapper v2.2 (https://cge.cbs.dtu.dk/services/MGmapper/) [19]. Paired-end reads from each metagenomic sample were mapped against several following databases composed of genome sequence data obtained from Genbank (http://www.ncbi.nlm.nih.gov/genbank/) and other resources (Analysis conducted from June 2016) (S1 File, S1 and S2 Tables).

#### Determination of the abundance of bacteria, viruses, and parasites

Of the bacteria, viruses, and parasites detected, we focused on pathogens of relevance to the global burden of infectious diseases [1,2,20–24]. The read abundance data were visualized using ggplot2 [25] and heatmap plotting systems for R [26]. To account for differences in sequencing depth between samples and to remove the influence of variation in bacterial/human reads, the following transformations were implemented: Bacteria and parasite mapped reads were shown as reads per million (RPM), calculated as (the number of reads mapped to a specific taxonomic group / total number of reads in the sample) *10^6^. For the viruses, the viral read count per million (VRPM) was calculated by normalizing the read count for each specific virus relative to the total viral read count for each sample as follows: (read count virus A/total viral read count)*10^6^. Several different algoritms have been developed to assist in outbreak detection, however, for simplicity we identified significant increases in abundance of individual pathogens during the study period. An upper limit was calculated as the mean read abundance plus 1.96 times the standard deviation [27]. Cases, where the observed weekly number of reads for specific bacteria, viruses or AMR genes were above the upper limit, these were defined as an “upsurge” of a sudden occurrence; all of those cases were excluded from the recalculation of the average and the upper limit (S3–S8 Tables). The number of *S*. Typhi reads at both clusters 9 and 10 in week 28 were removed from the calculation due to the spiked samples.

In addition, the relative abundance of the top 20 most abundant bacterial species were determined independent of their known relevance to infectious diseases (S9 Table).

#### Determination of the abundance of antimicrobial resistance

To calculate relative abundance of AMR genes (S10 Table), the raw counts (S11 Table) for each reference gene were converted to fragments per kilobase of transcript per million mapped reads (FPKM) before summing to gene class level as previously described [28]. For gene-level abundances, reference-level counts were summed to gene-level and were then transformed using regularized log transformation in DESeq2 as previously described [28].

Abundances were visualized in heatmaps produced using the R package ‘pheatmap’. For the AMR heatmaps, Euclidean distances between AMR features were clustered using complete linkage to draw dendrograms. For visualization, each AMR feature was transformed to Z-scores to enable easy between-sample comparison within a single AMR feature.

## Results

### Surveillance data and laboratory-confirmed cases

**Fig 2:**
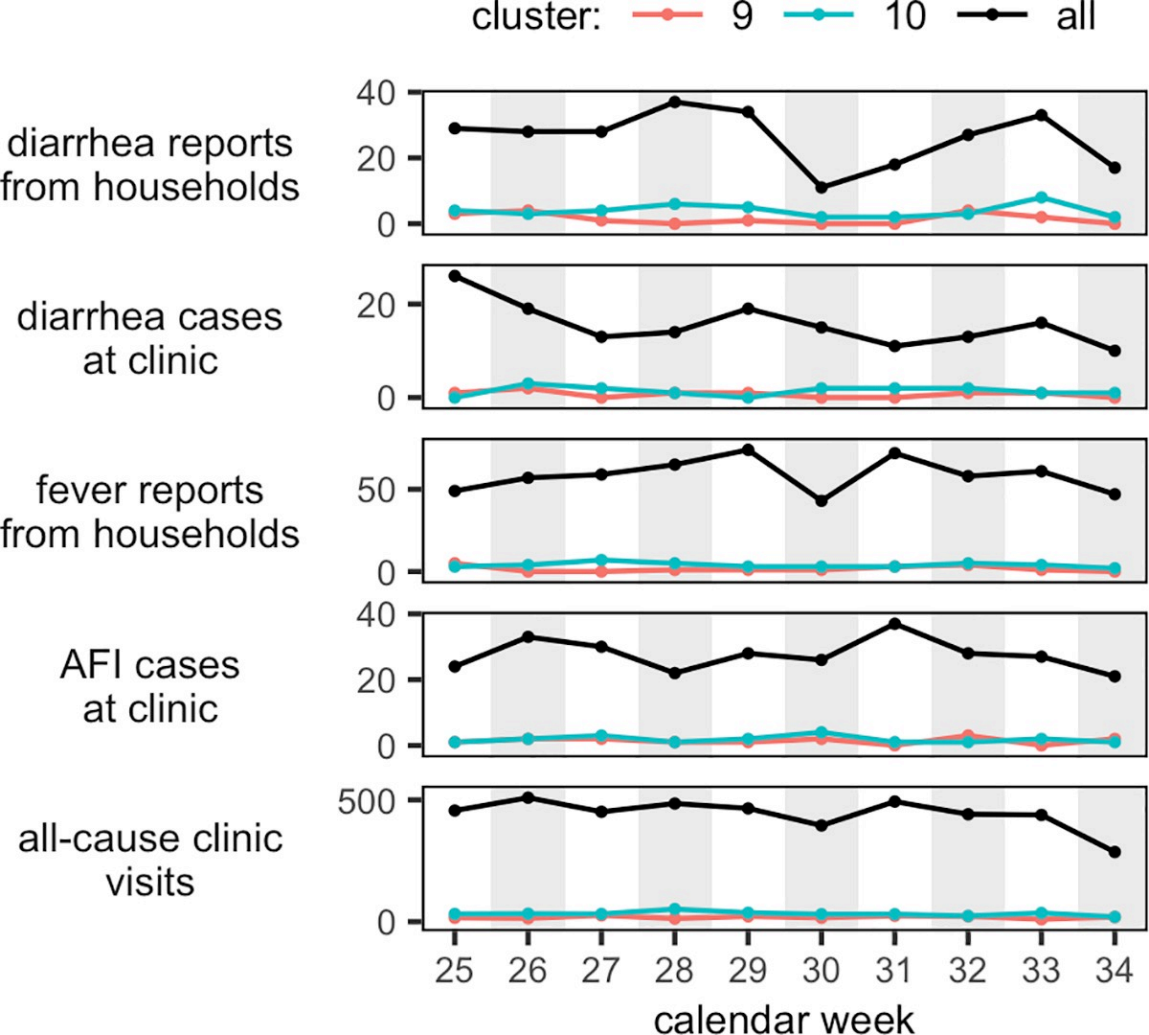
Data from the PBIDS from the Kibera clusters 9 and 10 in the period from June 18, 2014 (week 25) to August 20, 2014 (week 34). All cluster: black; cluster 9: red; cluster 10: green; X-axis is cases.

For cluster 9, cluster 10, and all clusters combined, a few weekly diarrhea and fever cases reported from the household surveillance as well as AFI cases, diarrhea cases and the all-cause clinic visits from the clinic data are presented in Fig 2. For all clusters, the highest number of household diarrhea cases occurred in weeks 28 (n = 37), 29 (n = 34), and 33 (n = 33), while the highest number of household fever cases were in weeks 29 (n = 74) and 31 (n = 72). For all clusters, the highest number of clinic diarrhea cases occurred in week 25 (n = 26), clinic fever cases in week 31 (n = 37), and all-cause clinic visits in week 26 (n = 509).

Very few bacterial pathogens were detected by culture of stool obtained from diarrhea cases from residents of clusters 9 and 10 visiting the clinic during the study period. One *Shigella flexneri* infection was detected the 17^th^ of August 2014 (week 34) (S12 Table).

### Genomic analysis

#### Sequencing data from DNA purification

An average of 10% and 9% of the reads mapped to the complete and draft bacterial genome databases, respectively. On average, 76% of the reads did not map to any of the queried reference databases. Rarefaction curves indicated that acceptable depth was obtained for bacteria and resistance genes, whereas greater depth could be desired for virus and parasites (S1 File). Summary mapping and read counts information can be found in S2 and S9 Tables.

#### Abundance of selected bacterial pathogens

**Fig 3:**
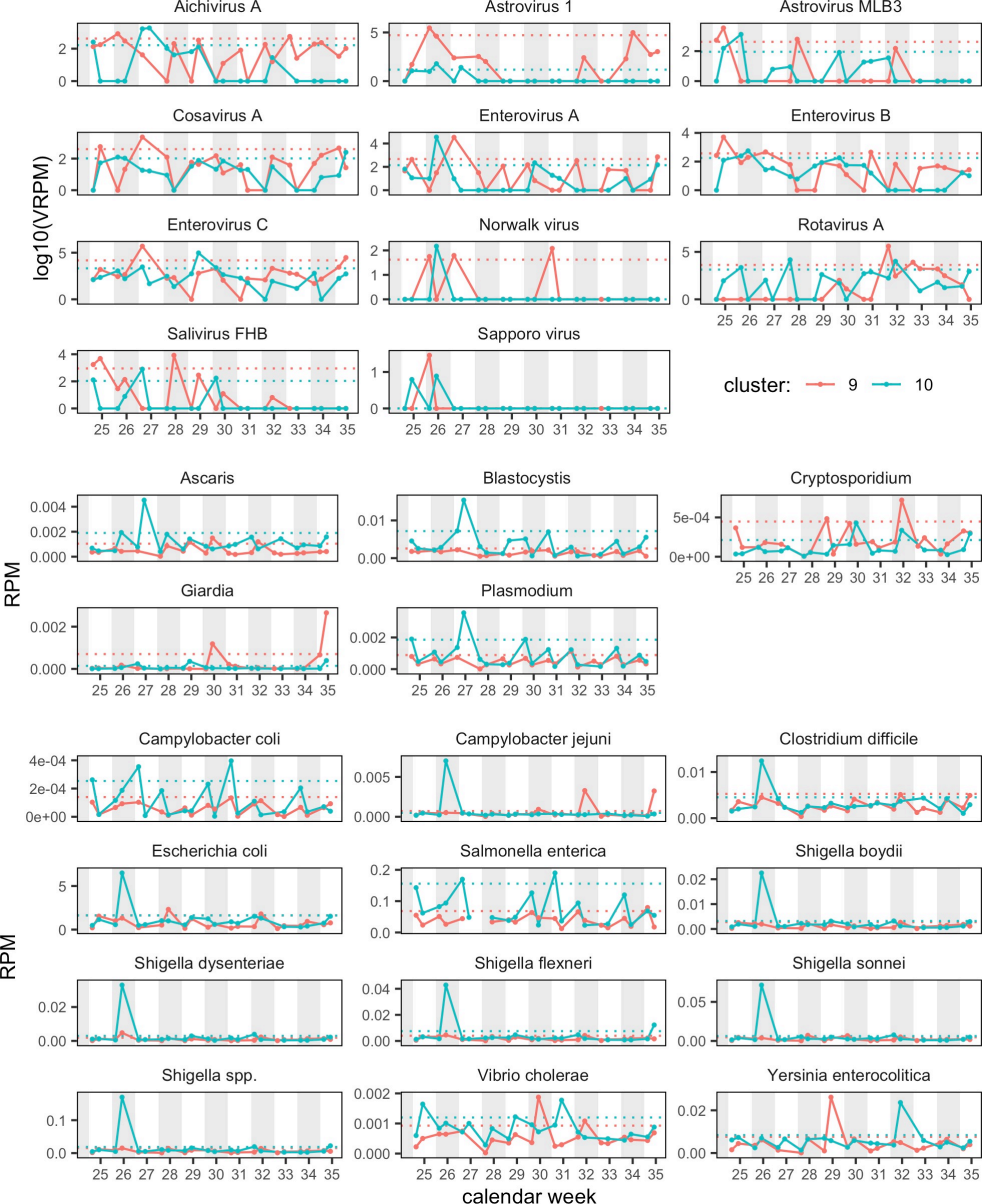
Relative read abundance (in RPM: Reads per million) of 27 human pathogens, (10 viral, 5 parasites, 12 bacterial) in sewage from Kibera. Red: cluster 9; blue: cluster 10. The dotted horizontal lines show upper limits for each cluster. Note that viral data are shown on the logarithmic scale (log10). Note that scale is individual for each pathogen.

A large variation in bacterial read abundances was observed over time with several peaks above the calculated upper limit, especially around weeks 25–27 and week 32 (Fig 3, S1 Fig, S7 and S8 Tables).

A high read abundance of *Shigella* spp. was observed in cluster 10 on Wednesday in week 26 with all four species exceeding the calculated upper limit (Fig 3). Similarly, bacterial read abundance of *Shigella* spp. was observed in cluster 9 on Wednesday in week 26 also exceeding the calculated upper limit but in lower read abundance compared with cluster 10 (Fig 3).

Other bacterial pathogens were also found to exceed the upper limit at week 26 in cluster 10, including *E*. *coli*, *Campylobacter* spp. and *Clostridium difficile* (Fig 3). No concurrence between the clusters was observed in the weeks where significantly higher read abundances to *Vibrio cholerae* and *Yersinia enterocolitica* exceeded the upper limit (Fig 3). None of the significantly higher increases were reflected in the PBIDS data (S12 Table).

The spiked sensitivity test with *S*. Typhi (collected from clusters 9 and 10 in week 28) lead to a large change in the relative abundance of *S*. *enterica* (from 0.04% to 86% of all sequencing reads within seven days), and the presence of *S*. Typhi was later confirmed with the identification of 238.439 unique reads to *S*. Typhi str. P-stx-12 (CP003278.1) (S9 and S14 Tables).

#### Abundance and clustering of selected antimicrobial resistance genes and classes

**Fig 4:**
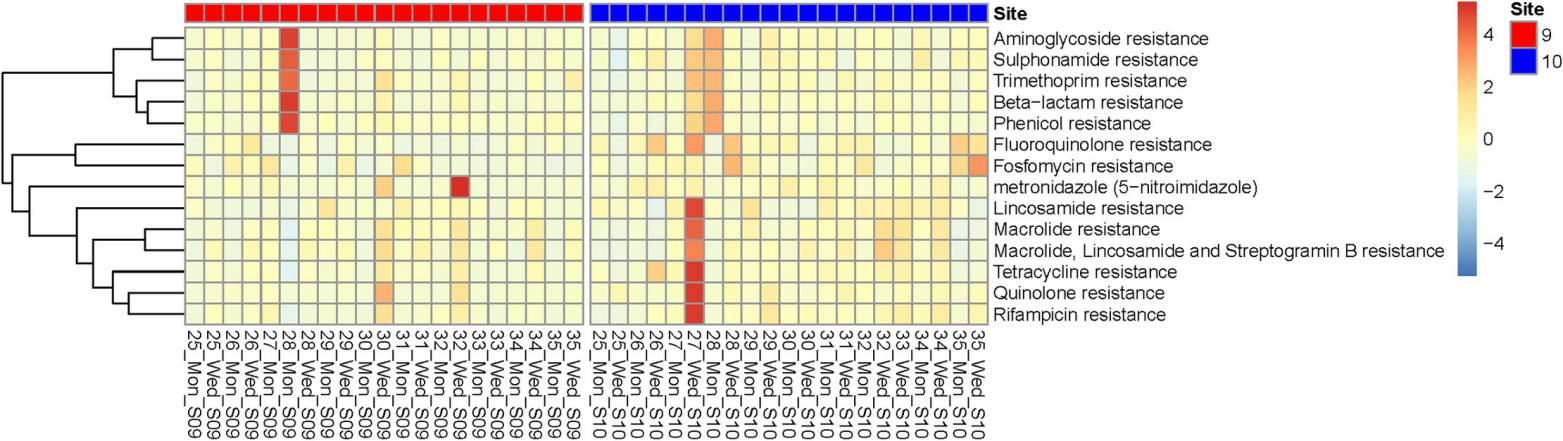
Heatmap showing changes in AMR abundance over time in clusters 9 and 10. Relative abundance (FPKM) was calculated for AMR at drug class level. AMR classes (rows) are clustered according to co-abundance using complete linkage clustering of Euclidean distances. Data were mean-standardized (Z-scores) within each drug class, enabling within-class, cross-sample interpretation. Colors represent log (ln) transformed relative abundances (FPKM).

The relative abundance of AMR genes was very low during the study period with only a few weekly point increases, for example, the high relative abundances on Wednesday of week 26 in cluster 10 of both tetracycline (*tet*A and *tet*40) and fluoroquinolones (Fig 4, S3 Fig, S10 and S11 Tables). In addition, a high relative abundance of metronidazole resistance genes was observed at cluster 9 on Wednesdays of weeks 30 and 32 (Fig 4). A sudden and considerable increase in the relative abundances of a number of AMR genes and classes was observed on Wednesday of week 27 in cluster 10 (Fig 4, S3 Fig and S10, S11 and S15 Tables). However, no corresponding increase in any of the bacterial pathogens was observed at the same time point (S4 Fig and S9 Table).

The spiked *S*. Typhi had several resistance genes, leading to high relative abundances of aminoglycoside (*str*A, *str*B), sulphonamide (*sul*1, *sul*2, *sul*3), trimethoprim (*dfr*A), beta-lactam (*bla*_TEM_, and phenicol (*cat*A) resistance genes in the samples from Monday of week 28 (Fig 4 and S3 Fig).

#### Abundance of selected viral pathogens

The most abundant viruses in the sewage samples were plant pathogens and members of the family *Virgaviridae*, but bacteriophages of the families *Siphoviridae* and *Podoviridae* were also found consistently (S5 Fig). Several human viral pathogens were detected during the course of the study, sometimes leading to substantial shifts in the sewage virome (S5 Fig). These viruses included rotavirus A, enterovirus C, mamastrovirus 1, enterovirus A, enterovirus B, astrovirus MLB3, and norovirus, and they all had their highest reads counts in weeks 25, 26, 30, and 31, respectively (Fig 3, S3 and S4 Tables). Putative enteric pathogens such as salivirus FHB, aichivirus A, and cosavirus A also showed significantly higher read abundances exceeding the upper limit in week 26 ± 1 week in one or both research clusters (Fig 3).

#### Abundance of selected parasitic pathogens

*Giardia* spp., *Plasmodium* spp., *Ascaris* spp., and *Blastocystis* spp. were the most abundant parasites throughout the study period. All observed parasites fluctuated during the study period with multiple increased abundances (Fig 3 and S5 and S6 Tables). *Cryptosporidium* spp. had significantly higher read abundances in weeks 29 and 30 as well as week 32 in cluster 9 and on the Wednesday of week 30 and week 32 in cluster 10 (Fig 3). Similarly, *Giardia* spp. also showed significant increases several times during the study period in both research clusters (Fig 3). Only one significant increase in read abundances exceeding the upper limit was observed with *Blastocystis* spp. on the Monday of week 27 (Fig 3).

#### Abundance of the bacteriome

Overall, the relative abundance of the most common bacterial species such as *Streptococcus* spp. *Klebsiella* spp. and *Enterobacter* spp. etc. were observed to fluctuate across the study period and among the clusters (S4 Fig). Besides the organisms identified as increasing in abundance and known for their ability to cause human illness, such as *E*. *coli*, a few bacterial genera were among the most abundant and were present once during the study period. This included *Burkholderia* spp. and *Brucella* spp. which were observed with a higher abundance on Monday of week 26 in cluster 9 (S4 Fig). This coincided with other bacterial genera of higher abundances in the same week such as *Shewanella* spp., *Salmonella* spp., *Bacillus* spp., *Campylobacter* spp. and *Flavobacterium* spp.

#### Epidemiological associations

There was a very low number of weekly reported counts of fever and diarrhea cases (from both household and clinic), all-cause clinic visits, and enteric pathogens identified from diarrhea cases presenting to the clinic. Thus, it was not possible to detect any statistically variations based on the conventional surveillance data (Fig 2, S12 Table). We did however, observe a tendency of the higher abundances of bacterial, viral, and parasitic pathogens as measured by metagenomics to coincide with reported increases in household diarrhea and subjective fever cases as well as clinic visits due to diarrhea and AFI.

## Discussion

Metagenomics has the potential to provide complete taxonomic and functional profiles of environmental and human microbiomes and resistomes [7–10]. Here, we evaluated and demonstrated that it is possible using a metagenomic approach to monitor pathogens circulating in a population residing in two surveillance clusters of the Kibera settlement. Thus, it was possible to identify and quantify human pathogens (bacteria, viruses, and parasites) and AMR determinants from sewage samples. The metagenomic surveillance approach provided a high resolution of microorganisms compared with traditional disease surveillance, and it could lead to new applications involving strategic genomics-based testing of environmental specimens in disease hot spots replacing the currently limited conventional surveillance.

Our analysis revealed a significant increase in the read abundance of *Shigella* spp. in week 26 from clusters 9 and 10, likely associated with an increase in tetracycline and fluoroquinolones resistance genes commonly present in this genus [14]. The concurrence of significant higher bacterial read abundances of *Shigella* spp. being present in both clusters could indicate a possible increased presence of the pathogen in the community of these two clusters (Fig 3, S7 and S8 Tables). We identified the *tet*40 gene, which is an efflux-type resistance gene encoding a predicted membrane-associated protein with 42% amino acid homology to the *tet*A gene. Previously, *tet*40 has been detected in *Clostridium* spp., we however, could not detect a coinciding read abundance with *Clostridium* spp. [29]. *Shigella* spp. is endemic in sub-Saharan Africa with a high incidence in Kibera affecting 1 in 200 people annually [14]. Other increases based on read mapping were observed for *Campylobacter* spp., *V*. *cholerae*, and *Y*. *enterocolitica*, which similarly have been shown to cause substantial disease burden in sub-Saharan Africa [1]. From a public health perspective, detection of endemic pathogens (e.g. *Shigella* spp., *Campylobacter* spp., and *Y*. *enterocolitica*) from sewage is most relevant if the levels represent an increase from baseline. For non-endemic, outbreak-prone pathogens, such as *V*. *cholera* or polioviruses, any detection of circulation is of interest to public health officials.

A number of human viral pathogens were observed during the study period. Among those, *rotavirus* A, has been shown to be the leading cause of severe gastroenteritis in children in Kenya as well as being responsible for an estimated 4,000 deaths among children <5 years of age in 2013 [2,30]. Rotarix, a live attenuated rotavirus vaccine [2,31–33], was introduced in Kenya in July 2014, in the midst of the study period. The rotavirus abundance observed in this study could reflect circulating rotavirus. Read abundances could however also reflect vaccine virus as it is possible for live vaccines to be shed in feces. Metagenomics surveillance approaches for pathogens in sewage must be able to distinguish between pathogenic and vaccine strains, including rotavirus and oral cholera vaccines, in order to provide useful public health information.

Interestingly, the PBIDS data did not show a peak in diarrheal cases during a time with significantly high read abundances for astrovirus 1, norovirus and sapovirus observed in both surveillance clusters in weeks 26 and 27. These viruses have recently been shown to be major contributors to gastroenteritis in Kibera, both alone and as coinfections [34]. Astrovirus MLB3 was another viral pathogen observed that temporally increase and is suspected to cause gastroenteritis. Astrovirus MLB3 has previously been shown to be the most prevalent astrovirus type in Kenya [35]. However, as no association with gastroenteritis was found in the study of Meyer et al. (29), it is unclear to what extent Astrovirus MLB3 contributed to the diarrheal cases detected at the clinic. Salivirus FHB, aichivirus A, cosavirus and human parechovirus were detected throughout this study and are all putative causes of gastroenteritis [36,37]. Interestingly, greater read depth would have been beneficial to the metagenomics analysis of virus and parasite but resulted in a greater discrepancy between the number of diarrheal cases and a higher abundance of virus. Of note, although a variety of viral pathogens was observed in the virome of the urban sewage, it is difficult to establish the disease associations of these pathogens. Many viral pathogens can be found in the stool of healthy individuals and are not necessarily the cause of diarrheal illness. Nonetheless, the broad range of enteric pathogens detected highlights the need for sanitation improvements in urban settlement cities like Kibera.

In this study, we found *Giardia* spp., *Plasmodium* spp., *Ascaris* spp., and *Blastocystis* spp. to be the most abundant parasite species throughout the study period. *Giardia* spp. is a very common intestinal parasite and is frequently reported from low- to middle-income countries, especially among children [38]. A study investigating the prevalence of intestinal parasites in children from another urban settlement of Nairobi, demonstrated that 26% of the tested children with diarrhea were positive for at least one intestinal parasite, supporting our findings and calling for an increased focus on intestinal parasites [39,40].

A number of limitations should be noted for the current study. The metagenomics analysis is still evolving but currently it is very difficult to link an identified pathogen with a AMR gene without a longer read technology applied or using metagenomics assemblies which is time and computational demanding.

Environmental factors, such as rainfall, could affect the number of pathogens detected, as the waste could either be accumulated in the sampling sites or drain away. These environmental factors along with the procedure of sewage sampling makes the surveillance strategy vulnerable to stochastic events. Due to the small number of urban sewage samples, it was difficult to detect outbreaks as no prior information concerning baseline pathogen abundance values exist from these sites. As viral tests were not routinely performed on stools collected through PBIDS surveillance during this study period, the presence of viruses among diarrhea cases could not be confirmed, although the metagenomic findings were in line with previous studies [34]. The PBIDS surveillance datasets were limited in the number of reported illnesses, diarrhea, AFI, subjective fever and clinic visits at the household level, making direct comparison between population-based disease surveillance and the relative abundances observed in metagenomics sewage surveillance very difficult and challenging. This might be improved if the household visits were conducted more often than once every two weeks. It is, however, interesting that several detected bacterial and viral pathogens in the metagenomics analysis coincide with increased numbers of diarrheal cases despite the few isolated bacteria from the clinic. Although this observation is intriguing, it could merely be a spurious correlation. Future investigations, incorporating longer periods of time for observation and sample collection and with a greater number of cases and more extensive or comprehensive clinic laboratory testing, should be conducted to better elucidate the findings of our study.

## Conclusion

This descriptive analysis of the metagenomic data obtained from urban sewage illustrates the potential for this method to be used for future public health disease surveillance in challenging settings and may even serve as predictors for increases in diarrheal cases and clinical visits, as well as increased risk of exposure to specific pathogens from wastewater. This work represents a proof of concept study and suggests that metagenomics have a high surveillance sensitivity, and may as such become a valuable supplement for clinical and syndromic surveillance of large, urban populations, where early recognition of potential outbreaks is crucial for timely outbreak containment.

## Supporting information

S1 File Full description of the method applied. (DOCX)

S1 Fig Rarefaction curves of bacterial and associated AMR genes (ResFinder), virus and parasitic species. For each sample, the mapped reads were randomly subsampled to varying levels (x-axis) to determine the unique number of genes or species hit (y-axis). Each line thus shows the trajectory for a single sample with a horizontal plateau indicating saturation.
(TIF)

S2 Fig The relative abundance of selected bacterial, viral, and parasitic pathogens based on normalized read counts by location and week. **Note that scale is individual for each pathogen.** The heatmap of normalized abundance is presented in log10 scale from blue (low) to red (high) whereas white indicate absence.
(TIF)

S3 Fig Heatmap showing changes in AMR gene abundance over time in clusters 9 and 10. Relative abundance (DESeq2 regularized log, ‘rlog’) was calculated for AMR genes, which adjusts for sequencing depth and minimize sample differences caused by genes with low counts which are very sensitive to random sampling effects. AMR genes (rows) are clustered according to co-abundance using complete linkage clustering of Euclidean distances. Data were mean-standardized (Z-scores) within each AMR gene, enabling within-gene, cross-sample interpretation.
(TIF)

S4 Fig Aggregation of the top 20 bacterial genera observed per week. Heatmap showing relative abundance changes for the bacterial genera among the 20 most abundant in any sample. Abundances are expressed as Z-scores based on reads per million (RPM), enabling easy within-genus, cross-sample interpretation.
(TIF)

S5 Fig Stacked bar chart of viral families. (TIF)

S1 Table Name of taxonomic databases including the number of included species. (XLSX)

S2 Table Number of reads mapping to the different taxonomic databases and species. (XLSX)

S3 Table Viral abundances cluster 9. (XLSX)

S4 Table Viral abundances cluster 10. (XLSX)

S5 Table Parasitic abundances cluster 9. (XLSX)

S6 Table Parasitic abundances cluster 10. (XLSX)

S7 Table Bacterial abundances cluster 9. (XLSX)

S8 Table Bacterial abundances cluster 10. (XLSX)

S9 Table Number of reads per top 20 most abundant pathogen per sample. (XLSX)

S10 Table Rlog-transformed counts (relative abundance) of raw counts of antimicrobial resistance genes. (XLSX)

S11 Table Number of raw counts of antimicrobial resistance genes aggregated on gene level. (XLSX)

S12 Table Number of diarrhea cases, acute febrile illness, and fever cases reported in households during optimal recall period and clinic visits for clusters 9, 10 and all clusters based on PBIDS data. (XLSX)

S13 Table Number of reads per sample and QC score. (XLSX)

S14 Table Read counts related to *Salmonella* Typhi reference genomes. (XLSX)

S15 Table FPKM-values per antimicrobial resistance classes. (XLSX)
