# Supplementary material for: Pathogen surveillance in the informal settlement, Kibera, Kenya, using a metagenomics approach
Source: PLoS One. 2019 Oct 10;14(10):e0222531. doi: 10.1371/journal.pone.0222531 (PMC6786639; doi:10.1371/journal.pone.0222531)
Supplement: S1 File — (DOCX) [file pone.0222531.s001.docx]

**Supplementary Text S1: Methods**

*Ethics*

This study was conducted in accordance with the Danish Act on scientific ethical treatment of health research (Journal no.: H-14013582) and fulfills the requirements of the Nagoya Protocol. Data from the Population-Based Infectious Disease Surveillance (PBIDS) platform were collected under a protocol approved by Kenya Medical Research Institute (KEMRI) and US Centers for Disease Control and Prevention (CDC). The staff of the Scientific and Ethics Review Unit (SERU) which is central to research in KEMRI, the authority responsible for surveillance in Kibera, found it unnecessary to require an institutional SERU review board approval to collect sewage samples. The samples were collected from open sewage runoffs draining the study area, as part of the ongoing active infectious disease surveillance system (SCC protocol number 1899).

Thus, collection of sewage samples did not involve any interaction with human subjects nor endangered or protected animal species.

*Site, sample collections, storage and shipment*

The surveillance is conducted in two (Gatwekera and Soweto) out of twelve villages in Kibera slum, in Nairobi, Kenya. Kibera is one of the largest settlements in East Africa and covers an area of approximately 0.4 km2. It is densely populated (~70,000 persons/ km2) with poor sanitation and a high burden of infectious diseases (1-3). There are ten geographic units referred to as clusters in the study area. Human fecal waste from households' latrines in the study area, flow into a network of open sewage runoffs draining the area. Samples were collected from the drainage ditches at points of confluence, in two areas with the highest surface flow accumulation. Sewage samples were collected from clusters 9 (latitude/ longitude: -1.314199/ 36.78492) and 10 (latitude/ longitude: -1.314704/ 36.78666), at two low elevated locations with an altitude of 1722.55 and 1721.59 of cluster 9 and 10, respectively (4).

Since 2005, CDC and KEMRI have jointly operated the PBIDS system, routinely collecting household- and clinic-level data (1). Household morbidity and health care usage data were collected every two weeks through home visits. Members with fever, respiratory illness or diarrhea during the home visits were advised to seek care at a centrally located Tabitha clinic which offered free medical care for acute illnesses. Patients with acute febrile illness (AFI) defined as measured axillary temperature ≥38.0 °C, respiratory syndrome defined as cough or difficult breathing plus one of IMCI danger signs or diarrhea defined as reported $\geq$3 loose stools in 24 hours (1), had their blood and/or stool samples collected for testing by culture methods (5)[13]. PBIDs participants are free to seek care at other private and public health care facilities in the area as well as traditional healers. Free medical care for acute illness is, however, offered at the Tabitha clinic which is located within a ~1Km radius of all PBIDs households.

*Cluster, sample collections, storage and shipment*

Urban sewage samples were collected from drainage ditches in each of the two clusters (9 and 10) that drain large parts of the surveillance area (Figure 1). Each Monday and Wednesday, 500 mL of sewage were collected from two spots of highest surface flow accumulation in the clusters, 47 and 34 for cluster 9 and 10, respectively, during the study period (June 16 to August 26, 2014), typically the ‘dry season’ in Nairobi, resulting in a total of 42 samples (4) (Figure 1).

Collected samples were kept in cooler boxes and transported to a KEMRI laboratory in the study area within 2 hours of collection. At KEMRI laboratory the samples were stored with no presentation at -80 C° and further shipped frozen without coolers in batches to the Technical University in Denmark for DNA extraction and downstream metagenomics analysis. All samples arrived still frozen to Technical University in Denmark.

Without the knowledge of the authors responsible for the analysis, samples taken at both clusters 9 and 10 in week 28 were spiked with a 1-µl culture of *Salmonella enterica* serovar Typhi (*S*. Typhi) to test the sensitivity of the sewage metagenomics approach.

*Sewage Sample Processing*

Sewage samples were thawed for 48 hours at 4° C before processing. Two-hundred ml of each sample was spiked with 10^6^ quantitative reverse transcription PCR (RT-qPCR) units of recombinant Mengovirus, vMC_0_, (ATCC VR-2310) and 25 ml of glycin buffer (pH 9.6, 0.3M glycine, 5.5% beef extract). Each sample was centrifuged at 8,000g for 30 min. and the supernatant was extracted for metagenomics testing for viruses while the pellet was tested for bacterial and parasitic DNA.

Genomic DNA (from bacteria, parasites and DNA viruses) was extracted from the samples using the QIAamp Fast DNA Stool mini kit as previously described (6) and was sequenced using Illumina HiSeq(bacterial and parasitic DNA) and MiSeq (DNA and RNA viruses).

The 200 ml of supernatant were initially filtered through a 0.45-µm PES membrane (Jet Biofil, Guangzhou, China) to remove bacterial and eukaryotic cells and the sample concentrated using overnight polyethylene glycol 8000 precipitation. The virus-containing pellet was resuspended in 850 µl of phosphate buffer saline and treated with OmniCleave endonuclease (Epicentre, Wisconsin, USA) to remove extracellular DNA/RNA (7). The sample was further purified using a 1:1 mixture of chloroform-butanol, to remove nucleases and inhibitors.

Viral RNA and DNA were co-extracted using the Nucleospin RNA XS kit (Macherey-Nagel, Düren, Germany). First strand cDNA synthesis was performed using the SuperScript® III First-Strand Synthesis SuperMix (Invitrogen, Carlsbad, California) and second strand DNA synthesis using Klenow Fragment exo-polymerase (Thermo Scientific, Waltham, MA, USA) (7). Double-stranded DNA products were PCR amplified using the HotStarTaq Master Mix Kit (Qiagen, Valencia CA, USA). PCR products were purified using the MinElute PCR Purification Kit (Qiagen, Valencia CA, USA) before sequencing. NGS library preparation was performed using the Nextera XT DNA Library Preparation kit (Illumina) and paired end sequenced (PE, 2 x 250bp) on the Illumina MiSeq platform using the MiSeq Reagent Kit v3. Initially, trimming and removal of adaptor sequences was done using cutadapt (8) with settings for minimum read length being 30 bp and a minimum Phred quality score of 30, to trim low-quality reads before adaptor removal (cutadapt parameter - quality-cutoff). Raw sequence data have been submitted to the European

Nucleotide Archive under study accession no.: PRJEB13833.

*Metagenomics analysis*

Metagenomics analysis

Bacteria, viruses, parasites, AMR genes within the samples were identified and quantified using MGmapper v2.2 (https://cge.cbs.dtu.dk/services/MGmapper/) (9). Paired-end reads from each metagenomic sample were mapped against the following databases composed of genome sequence data obtained from Genbank (http://www.ncbi.nlm.nih.gov/genbank/) and other resources as specified: 1) parasites genomes, 2) complete bacterial genomes, 3) draft bacterial genomes, 4) whole genome viruses, 5) viruses extracted from nucleotide database (Virus_nt), and 6) ResFinder (Analysis conducted from June 2016) (Supplementary Table S2 and S3). Rarefaction of the read mapping to the queried reference databases indicated an acceptable degree of saturation (Supplementary Figure S1), which suggests that a sufficient amount of data was generated.

*Determination of the abundance of antimicrobial resistance*

To calculate relative abundance of AMR (Supplementary Table S11), the raw counts (Supplementary Table S12) for each reference were converted to FPKM before summing to gene class level as previously described (10). For gene-level abundances, reference-level counts were summed to gene-level and were then transformed using regularized log transformation in DESeq2 as previously described (10).

Abundances were visualized in heatmaps produced using the R package ‘pheatmap’.

For the AMR heatmaps, Euclidean distances between AMR features were clustered using complete linkage to draw dendrograms. For visualization, each AMR feature was transformed to Z-scores to enable easy between-sample comparison within a single AMR feature.

*Determination of the abundance of bacteria, viruses, and parasites*

Of the bacteria, viruses, and parasites examined, we focused on pathogens of relevance to the global burden of infectious diseases (1;11-16). The read abundance data were visualized using ggplot2 (17) and heatmap plotting systems for R (10). To account for differences in sequencing depth between samples and to remove the influence of variation in bacterial/human reads, the following transformations were implemented: Bacteria and parasite mapped reads were shown as reads per million (RPM), calculated as (the number of reads mapped to a specific taxonomic group / total number of reads in the sample) *10^6^. For the viruses, the viral read count per million (VRPM) was calculated by normalizing the read count for each specific virus relative to the total viral read count for each sample as follows: (read count virus A/total viral read count)*10^6^. To identify significant increases in abundance of individual pathogens during the study period, an upper limit was calculated as the mean read abundance plus 1.96 times the standard deviation. Cases where observed weekly numbers of reads were above the upper limit were defined as an “upsurge” of a sudden occurrence; all of those cases were excluded from the recalculation of the average and the upper limit (Supplementary Tables S4, S5, S6, S7, S8, and S9). The number of *S*. Typhi reads at both clusters 9 and 10 in week 28 were removed from the calculation due to the spiked samples.

In addition, the relative abundance of the top 20 most abundant bacterial species were determined independent of their known relevance to infectious diseases (Supplementary Table S10).

Reference List

(1) Feikin DR, Olack B, Bigogo GM, Audi A, Cosmas L, Aura B et al. The burden of common infectious disease syndromes at the clinic and household level from population-based surveillance in rural and urban Kenya. PLoS One 2011 January 18;6(1):e16085.

(2) Scott AA, Misiani H, Okoth J, Jordan A, Gohlke J, Ouma G et al. Temperature and heat in informal settlements in Nairobi. PLoS One 2017 November 6;12(11):e0187300.

(3) Njuguna HN, Cosmas L, Williamson J, Nyachieo D, Olack B, Ochieng JB et al. Use of population-based surveillance to define the high incidence of shigellosis in an urban slum in Nairobi, Kenya. PLoS One 2013;8(3):e58437.

(4) Akullian A, Ng'eno E, Matheson AI, Cosmas L, Macharia D, Fields B et al. Environmental Transmission of Typhoid Fever in an Urban Slum. PLoS Negl Trop Dis 2015 December 3;9(12):e0004212.

(5) Kiruki Silas. *Prevalence of multidrug resistant enteropathogenic bacteria causing diarrhea in Kibera community in Nairobi, Kenya*. Mageto O Kennedy NWBGSK, editor. 1 2, 24-30. 2006. *Journal of Tropical Microbiology and Biotechnology*.
Ref Type: Generic

(6) Knudsen BE, Bergmark L, Munk P, Lukjancenko O, Prieme A, Aarestrup FM et al. Impact of Sample Type and DNA Isolation Procedure on Genomic Inference of Microbiome Composition. mSystems 2016 October 18;1(5):e00095-16.

(7) van LM, Williams MM, Koraka P, Simon JH, Smits SL, Osterhaus AD. Human picobirnaviruses identified by molecular screening of diarrhea samples. J Clin Microbiol 2010 May;48(5):1787-94.

(8) Martin M. Cutadapt removes adapter sequences from high-throughput sequencing reads. EMBnet J 2011;17:10-2. 2016.
Ref Type: Generic

(9) Petersen TN, Lukjancenko O, Thomsen MCF, Maddalena SM, Lund O, Moller AF et al. MGmapper: Reference based mapping and taxonomy annotation of metagenomics sequence reads. PLoS One 2017 May 3;12(5):e0176469.

(10) R Core Team (2014). R: A language and enviroment for statistical computing. R Foundation for Statistical Computing 2014 January 1.

(11) Lozano R, Naghavi M, Foreman K, Lim S, Shibuya K, Aboyans V et al. Global and regional mortality from 235 causes of death for 20 age groups in 1990 and 2010: a systematic analysis for the Global Burden of Disease Study 2010. Lancet 2012 December 15;380(9859):2095-128.

(12) Zankari E, Hasman H, Cosentino S, Vestergaard M, Rasmussen S, Lund O et al. Identification of acquired antimicrobial resistance genes. J Antimicrob Chemother 2012 November;67(11):2640-4.

(13) Majowicz SE, Musto J, Scallan E, Angulo FJ, Kirk M, O'Brien SJ et al. The global burden of nontyphoidal Salmonella gastroenteritis. Clin Infect Dis 2010 March 15;50(6):882-9.

(14) Crump JA, Luby SP, Mintz ED. The global burden of typhoid fever. Bull World Health Organ 2004 May;82(5):346-53.

(15) Kotloff KL, Winickoff JP, Ivanoff B, Clemens JD, Swerdlow DL, Sansonetti PJ et al. Global burden of Shigella infections: implications for vaccine development and implementation of control strategies. Bull World Health Organ 1999;77(8):651-66.

(16) Murray CJ, Lopez AD. Alternative projections of mortality and disability by cause 1990-2020: Global Burden of Disease Study. Lancet 1997 May 24;349(9064):1498-504.

(17) Wickham H. ggplot2: Elegant Graphics for Data Analysis. Springer; 2009. 2016.
Ref Type: Generic
