# Supplementary figures and images for: Pathogen surveillance in the informal settlement, Kibera, Kenya, using a metagenomics approach
Source: PLoS One. 2019 Oct 10;14(10):e0222531. doi: 10.1371/journal.pone.0222531 (PMC6786639; doi:10.1371/journal.pone.0222531)

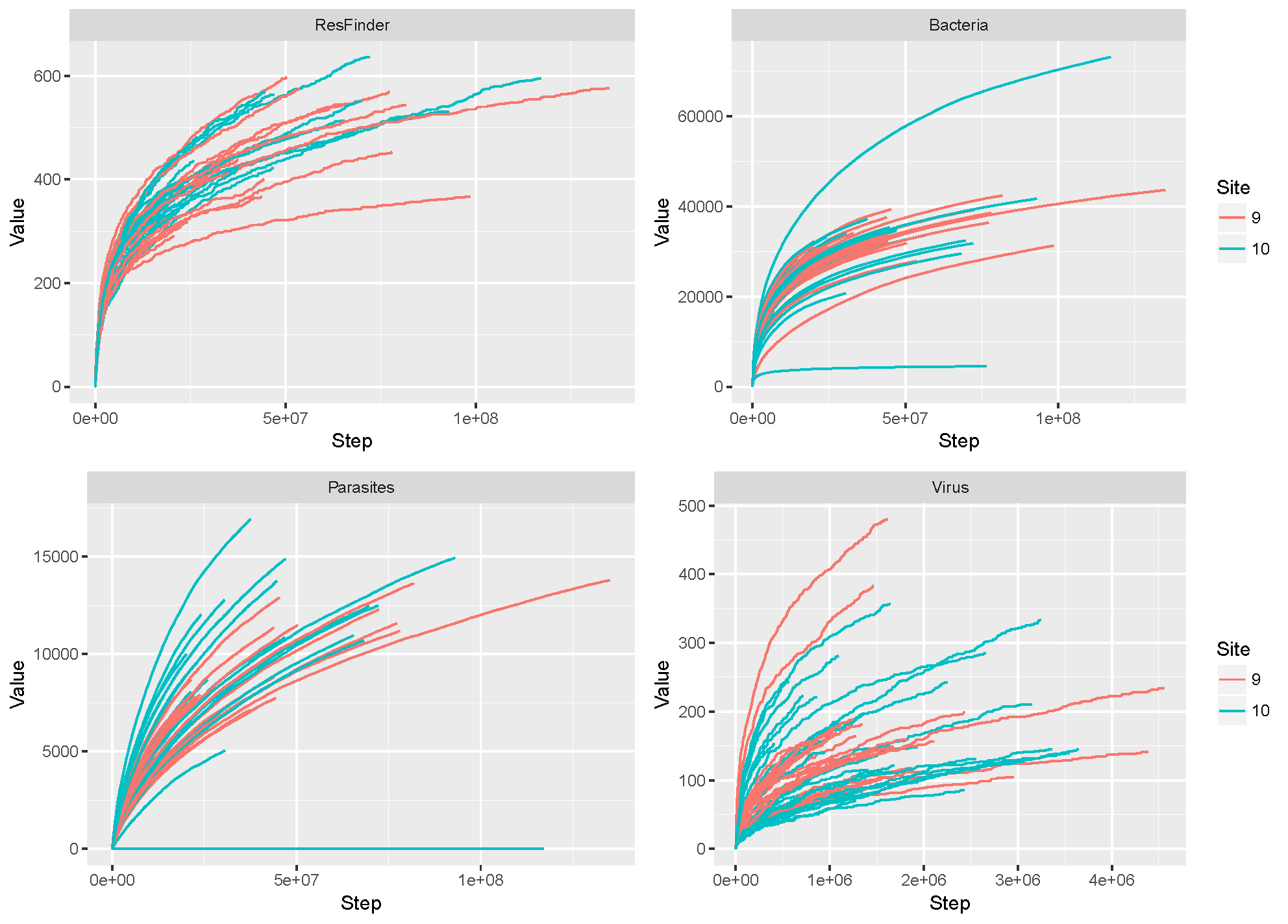

Supplement: S1 Fig — For each sample, the mapped reads were randomly subsampled to varying levels (x-axis) to determine the unique number of genes or species hit (y-axis). Each line thus shows the trajectory for a single sample with a horizontal plateau indicating saturation. (TIF) [file pone.0222531.s002.tif]

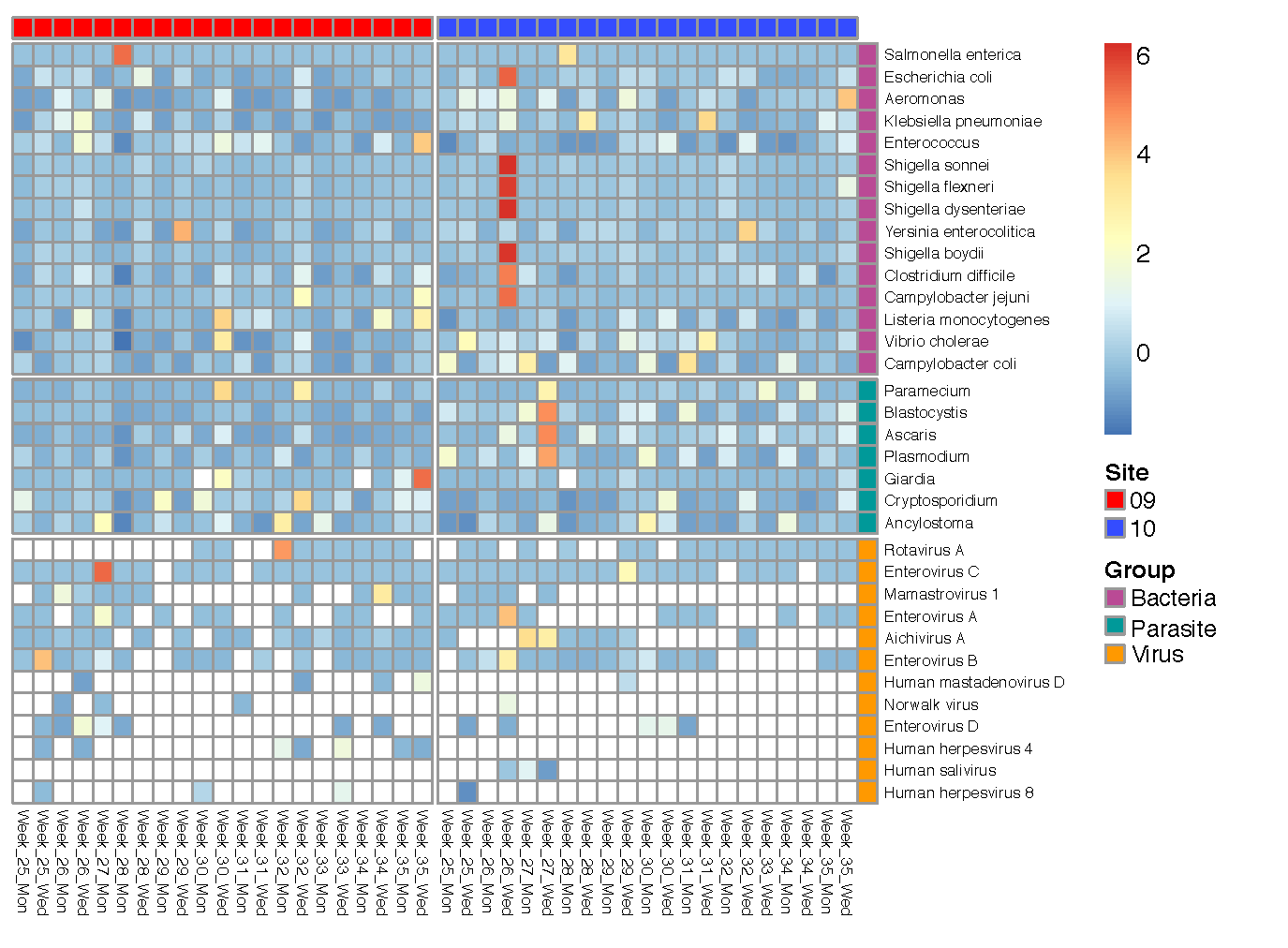

Supplement: S2 Fig — Note that scale is individual for each pathogen. The heatmap of normalized abundance is presented in log10 scale from blue (low) to red (high) whereas white indicate absence. (TIF) [file pone.0222531.s003.tif]

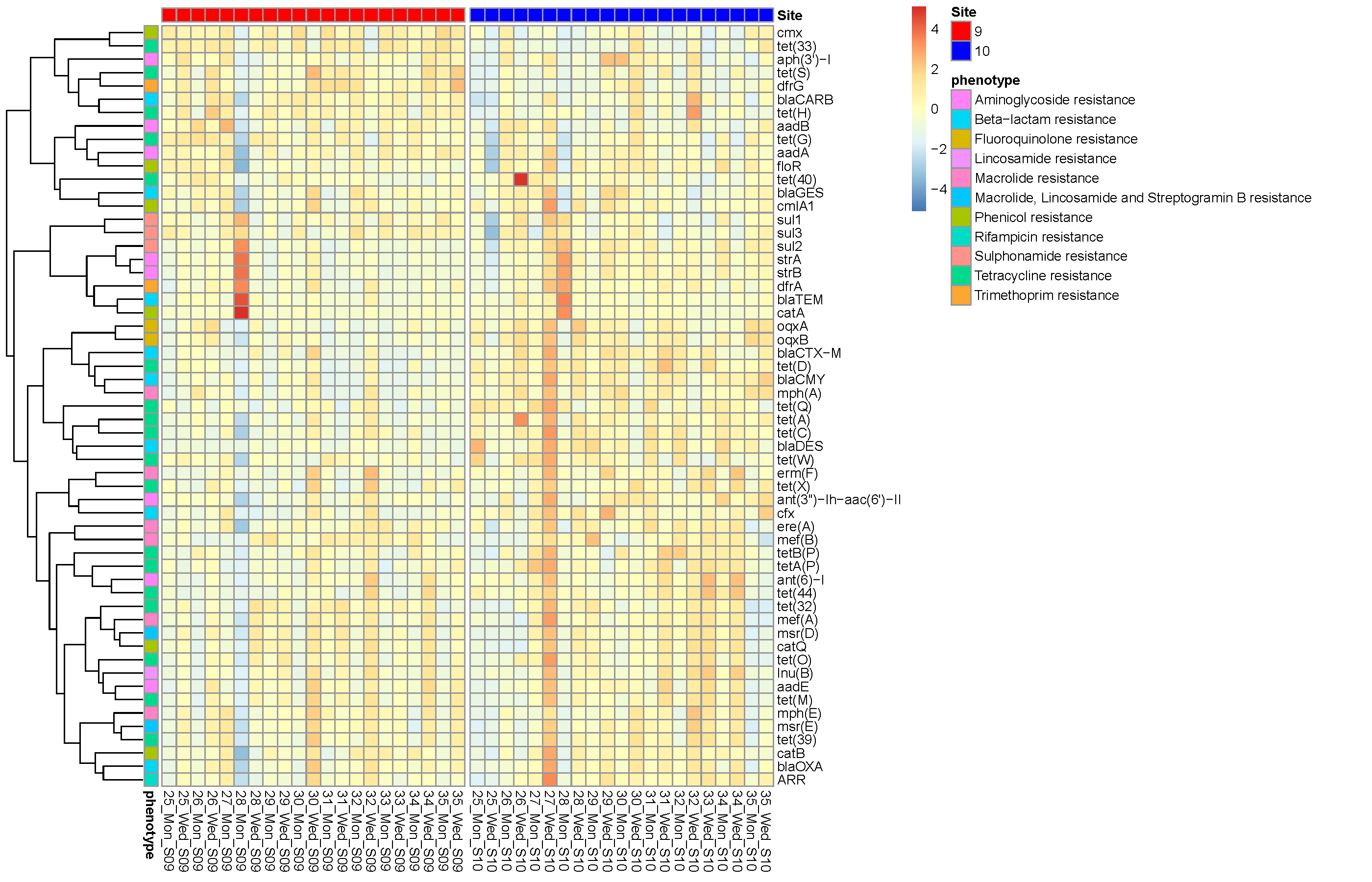

Supplement: S3 Fig — Relative abundance (DESeq2 regularized log, ‘rlog’) was calculated for AMR genes, which adjusts for sequencing depth and minimize sample differences caused by genes with low counts which are very sensitive to random sampling effects. AMR genes (rows) are clustered according to co-abundance using complete linkage clustering of Euclidean distances. Data were mean-standardized (Z-scores) within each AMR gene, enabling within-gene, cross-sample interpretation. (TIF) [file pone.0222531.s004.tif]

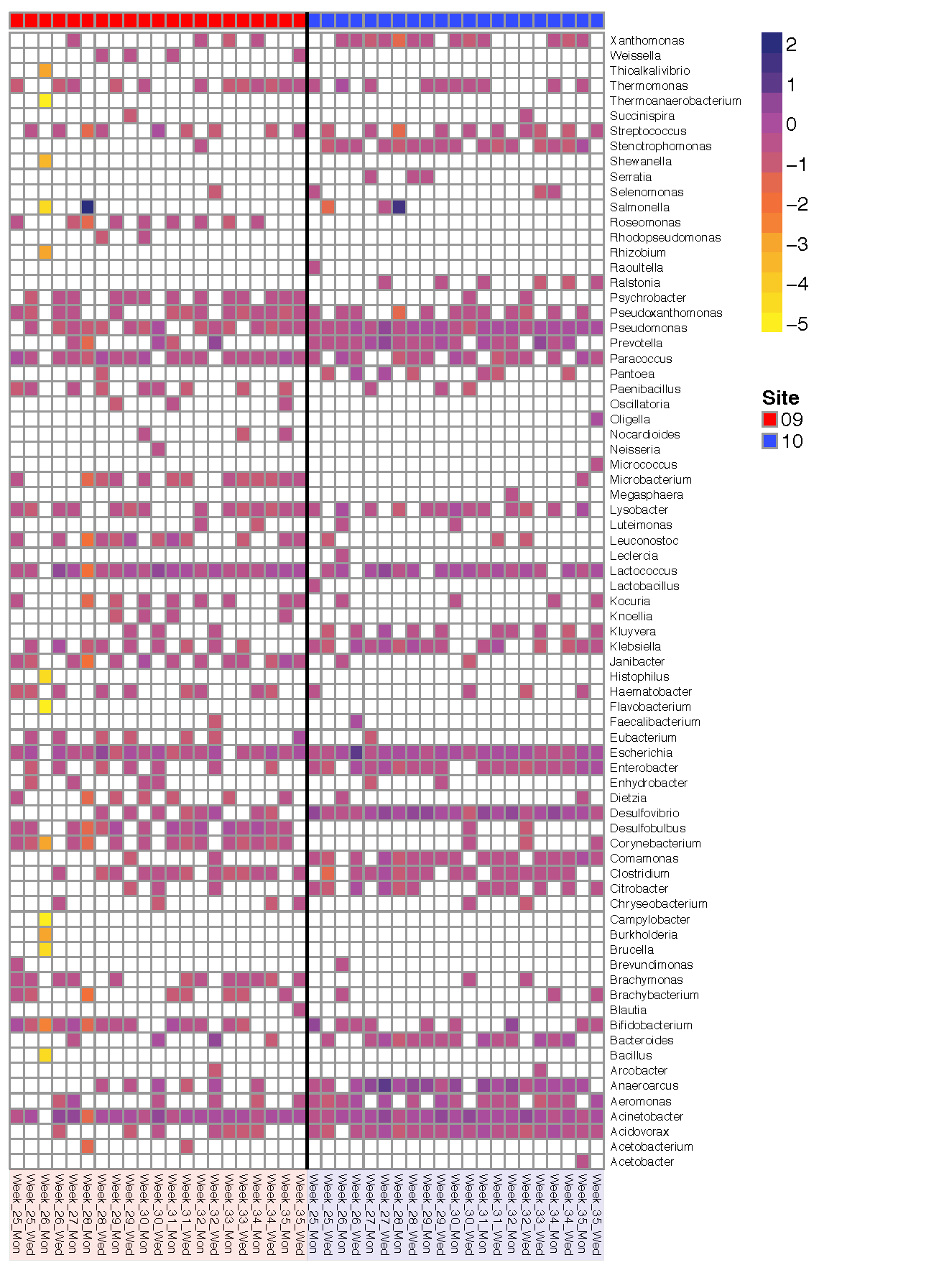

Supplement: S4 Fig — Heatmap showing relative abundance changes for the bacterial genera among the 20 most abundant in any sample. Abundances are expressed as Z-scores based on reads per million (RPM), enabling easy within-genus, cross-sample interpretation. (TIF) [file pone.0222531.s005.tif]

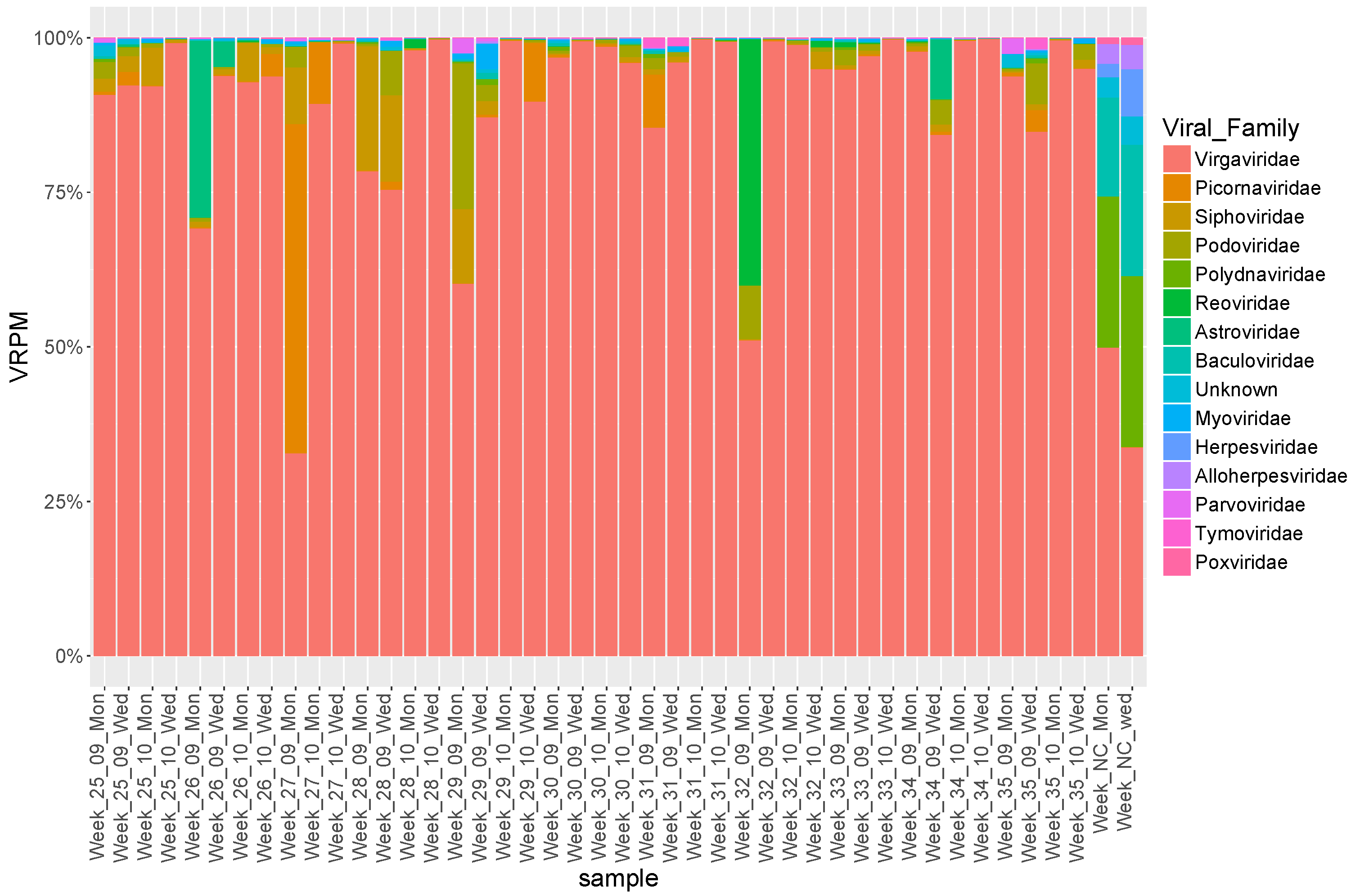

Supplement: S5 Fig — (TIF) [file pone.0222531.s006.tif]
